# Membrane transport proteins in human melanoma: associations with tumour aggressiveness and metastasis

**DOI:** 10.1038/sj.bjc.6605590

**Published:** 2010-03-16

**Authors:** N Walsh, S Kennedy, A M Larkin, D Tryfonopoulos, A J Eustace, T Mahgoub, C Conway, I Oglesby, D Collins, J Ballot, W S Ooi, G Gullo, M Clynes, J Crown, L O'Driscoll

**Affiliations:** 1 National Institute for Cellular Biotechnology, Dublin City University, Dublin 9, Ireland; 2 St. Vincent's University Hospital and Molecular Therapeutics for Cancer Ireland (MTCI), Dublin 4, Ireland; 3 MTCI, c/o National Institute for Cellular Biotechnology Building, Dublin City University, Dublin 9, Ireland; 4 SlidePath, Santry, Dublin 9, Ireland; 5 School of Pharmacy and Pharmaceutical Sciences and MTCI, Trinity College Dublin, Dublin 2, Ireland

**Keywords:** malignant melanoma, multiple drug resistance, MDR1/P-gp, MRP-1, immunohistochemistry

## Abstract

**Background:**

Malignant melanoma, generally described as incurable, is notoriously refractory to chemotherapy. The mechanisms contributing to this have not yet been defined and the contributions of drug efflux pumps, implicated in chemo-resistance of many other cancer types, have not been extensively investigated in melanoma.

**Methods:**

In this study, expression of multi-drug resistant (MDR1/P-gp and MRP-1) proteins was examined, by immunohistochemistry, in archival specimens from 134 melanoma patients. This included 92 primary tumours and 42 metastases.

**Results:**

On assessing all specimens, MRP-1 and MDR1/P-gp expression was found to be common, with the majority (81%) of melanomas expressing at least one of these efflux pumps. Although there is significant association between expression of these pumps (*P*=0.007), MRP-1 was found to be the predominant (67% of cases) form detected. *χ*^*2*^ analysis showed significant associations between expression of MRP-1 and/or MDR1/P-gp and the aggressive nature of this disease specifically increased Breslow's depth, Clark's level and spread to lymph nodes. This association with aggressiveness and spread is further supported by the observation that a significantly higher percentage of metastases, than primary tumours, express MRP-1 (91% vs 57% *P*<0.0001) and MDR1/P-gp (74% vs 50% *P*=0.010).

**Conclusion:**

The predominant expression of these pumps and, in particular, MRP-1 suggests that they may be important contributors to the inherent aggressive and resistant nature of malignant melanoma.

The incidence rate for cutaneous melanoma, which involves the malignant proliferation of melanocytes, is increasing faster than that of any other malignant disease, at an epidemic rate of 3% a year (Leiter and Gerbe, 2008; Gerbe and Leiter, 2009). Over the last century, melanoma has progressed from being considered as a rare cancer to a situation in which the lifetime risk of developing this disease is 1 in 50 in many Western populations (Giblin and Thomas, 2007), with major socioeconomic consequences as a result of the high mortality rates associated with this metastatic disease (Meyle and Guldberg, 2009). Currently, approximately 133 000 new cases of melanoma are diagnosed worldwide each year. The NCI's surveillance epidemiology and end results estimate that in the United States, in 2009 alone, 68 720 new cases of melanoma of the skin will be diagnosed and 8650 people will die from this disease.

Prognosis for patients with early-stage melanoma is very good and can often be cured by wide surgical excision. Metastatic disease, unfortunately, remains generally incurable and is largely resistant to current therapies, with a median survival time of 6 to 9 months from the time of diagnosis and a 5-year survival rate of 5.5% (Keller *et al*, 2006; Sarnaik and Weber, 2009). Biological therapies including IFN-α have shown a real, but minimal, effect in malignant melanomas (Pavlick *et al*, 2003) and the effects of IL-2, as a treatment for malignant melanoma, are not yet clearly defined. High-dose bolus IL-2 produces some long-term remissions and is available for highly selected individuals, but is not suitable for most patients (Mohr *et al*, 2003; Agarwala, 2009). Chemotherapeutic therapy for melanoma has been attempted with the many chemotherapeutic drugs. Single-agent chemotherapy, including dacarbazine, temozolomide, the nitrosoureas (BCNU, CCNU and methyl CCNU), the vinca alkaloids (vincristine, vinblastine and vindesine), cisplatin, paclitaxel and bleomycin, have been used. Many of these are relatively well-tolerated (including dacarbazine, which now has FDA approval for this indication), but are associated with response rates of only 5–20% (Bhatia *et al*, 2009; Seetharamu *et al*, 2009). Multi-drug chemotherapy regimes have resulted in objective response rates, but do not extend survival and are associated with greater toxicity (Nystrom *et al*, 2003; Pawlik and Sondak, 2003; Tas *et al*, 2003; Bhatia *et al*, 2009). The limited response of malignant melanoma to chemotherapy is apparently due to the inherent drug refractory-nature of this disease (Ichihashi and Kitajima, 2001; Colone *et al*, 2008). This has resulted in targeted therapies being fast-tracked through FDA approval, for example, Ipilimumab, which targets CTL4 and which is approved for metastatic and advanced melanoma. Such monoclonal antibodies are more expensive than traditional chemotherapy and so are an economic burden on many health systems. Unfortunately, they too may become of limited use because of the resistance nature of melanoma.

There is some, albeit limited, evidence that expression of genes encoding membrane efflux pumps (ABC transport proteins), including *MRP-1* (ABCC1) and multi-drug resistant 1(*MDR1)/P-gp* (ABCB1), may be associated with this chemo-resistance and so with the incurable nature of malignant melanomas. However, conflicting data exists on the details of the specific pump(s) involved. In a study of MRP-1 mRNA and protein expression in 40 melanoma cell lines, Berger *et al* (1997) reported sensitivity to a broad range of chemotherapeutic drugs to inversely correlate with MRP-1 expression. Furthermore, analysis of *mrp-1* and *mdr-1* mRNA expression in 18 specimens from eight melanoma patients' tumours showed *mrp-1* expression in the majority of pre-chemotherapy specimens, the levels of which were increased post-chemotherapy. *mdr-1* mRNA was, however, not detected in any of the specimens analysed (Ichihashi and Kitajima, 2001). In keeping with this, based on studies of 21 primary malignant melanomas and 37 metastases, Schadendorf *et al* (1995) reported 43% of both primary and metastatic tumours to express MRP-1, with a lack of detectable expression of MDR1/P-gp (except in one primary tumour) suggesting that the chemo-resistant nature of melanoma cells is unlikely to be mediated by MDR1/P-gp. Conversely, results from studies of human melanoma cultured cells (M14) suggested MDR1/P-gp, but not MRP-1, to be associated with the MDR phenotype (Molinari *et al*, 2000). In further studies of M14 cell line variants (specifically, MDR1/P-gp-positive drug-resistant M14 ADR compared with MDR1/P-gp-negative drug sensitive M14 cells), members of the same research group have more recently reported MDR1/P-gp to be linked with MAPK signalling and to be involved in melanoma cell migration and invasion (Colone *et al*, 2008).

As outlined above, although studies of melanoma cell line models support an association between MDR1/P-gp and/or MRP-1 and the chemo-resistant, generally incurable, phenotype of malignant melanoma, studies of the prevalence of MDR1/P-gp and MRP-1 in melanoma specimens have been very limited and conflicting. To determine the potential involvement of these drug efflux pumps in the clinical setting, here we report results from a retrospective study of both MDR1/P-gp and MRP-1 protein expression and their associations with patients' clinicopathological features (where possible) in 134 melanomas, including 92 primary tumours and 42 metastases.

## Materials and methods

### Patients

The patient group studied comprised 134 consenting patients with malignant melanoma. All patients were diagnosed at St Vincent's University Hospital (SVUH), Dublin, between 1975 and 2002 and approval to conduct this study was granted by the SVUH Ethics Committee. Formalin-fixed paraffin-embedded material was available for all patients. Representative 4 *μ*m sections of tissue blocks were cut using a microtome, mounted onto poly-l-lysine-coated slides and dried overnight at 37 °C. Slides were stored at room temperature until required.

### Immunohistochemistry

All steps were performed at room temperature unless otherwise indicated. Tissue sections were dewaxed in xylene (2 × 5 min), rehydrated in grading alcohols 100, 90 and 70% (2 × 3 min each), and placed in Tris Buffered Saline (TBS/0.05% Tween-20). Endogenous Peroxidase activity was quenched by placing tissue sections in 3% (v/v) H_2_O_2_/distilled water for 5-6 min. All slides were blocked for non-specific staining with 20% normal rabbit serum (Dako, Glostrup, Denmark, X-902)TBS for 20 min. Primary antibodies were applied to each specimen. Specifically, anti-MDR1/P-gp clone 6/1C ascites (which we developed and previously extensively characterised as specific for MDR1P-gp; Moran *et al*, 1997) were diluted 1:40 in TBS/0.05% Tween-20. Anti-MRP-1 PA28(6) antibody, (which we similarly developed and extensively characterised as specific for MRP-1; (Connolly *et al*, 2001)) was applied as a neat supernatant. Primary antibodies were incubated overnight at 4 °C. Samples were then washed (3 × 5 min) with TBS/0.05% Tween-20, followed by 30 min incubation with biotinylated secondary antibody diluted in TBS/0.05% Tween-20. Finally, following another 3 × 5 min. wash step, Vector SG substrate for peroxidase (Vector Laboratories, Peterborough, UK, SK-4700) was applied for approx. 10 min. All slides were washed (3 × 5 min.) in TBS/0.05% Tween-20. Tissue sections were then lightly counter-stained with nuclear fast red (Vector Laboratories, H-3403).

Following this, slides were dehydrated in 70, 90 and 100% grading alcohols (2 × 3 min.). Samples were then cleared in xylene and mounted in DPX (BDH, London UK). Positive control, normal kidney and lung tissue sections (using the same experimental conditions), and negative control samples (in which the primary antibody was replaced by 1 × TBS/0.05% Tween-20) were included in all experiments.

### Immunohistochemical scoring

MRP-1 and MDR1/P-gp immunohistochemical staining was scored (by the Consultant Pathologist, Prof. Susan Kennedy) according to the percentage of cells showing specific immunoreactivity. A semi-quantitative measurement was used in which the overall positivity of the tumour was assessed and a score of 1+ was given if up to 25% of cells showed MRP-1 or MDR1/P-gp positive staining, as relevant; a score of 2+ was given if ⩾25–<50% cells showed positive staining; a score of 3+, if ⩾50–<75% of cells showed positive staining; and a score of 4+, if ⩾75% of cells showed positive staining. Scores of 1 were considered as negative; scores > 1 were considered positive.

### Statistical analysis

Statistical analyses of the results were performed using SPSS 16.0 and InStat software packages. Descriptive statistics were used to summarise patient characteristics, and statistical analysis of the results was performed using Pearson's *χ*^*2*^ test to investigate relationships between MDR1/P-gp and MRP-1 expression and clinicopathological and histopathological findings. Kaplan–Meier survival curves were established and were subsequently checked using the log-rank, Breslow and Tarone-ware tests (*P*-values represent log-rank, unless otherwise indicated) to assess the prognostic significance of MDR1/P-gp and MRP-1 protein expression. The data were analysed as a whole and as primary and metastases cohorts, and, where relevant, the data were also censored at 5 years. A value of *P* <0.05 was considered to be statistically significant.

## Results

### Patient characteristics

This study involved analysis of tumours from 134 patients with metastatic melanoma. Eighty-three of the patients were female; 51 were male. The patients' age ranged between 22 and 104 years at the time of diagnosis (median age=59 years). A total of 92 of the specimens available for analysis were primary tumours; 42 were secondary metastases. Further clinical characteristics of the patients are summarised in Table 1.

### MDR1P-gp expression

**Figure 1:**
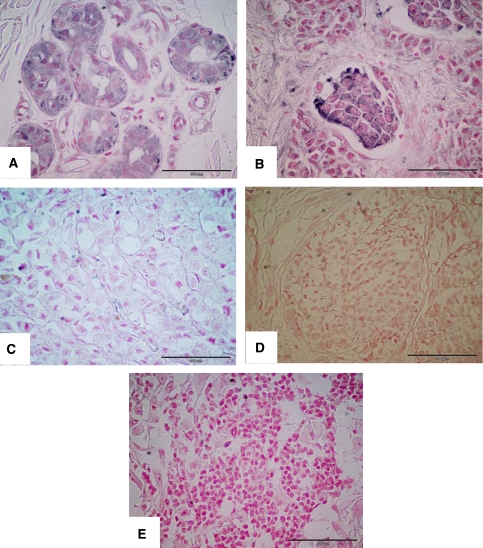
Immunohistochemical analysis of MDR1/P-gp and MRP-1 protein expression. Representative melanoma specimens showing strongly positive (4+3) MDR1/P-gp (**A**) and MRP-1 (**B**) staining (4+3). Images (**C**) and (**D**) show specimens that were negative for MDR1/P-gp and MRP-1 staining, respectively. Image (**E**) represents negative control (scale bar=100 *μ*m in all images; original magnifications of all photomicrographs × 40).

MDR1/P-gp specific staining was observed in 57.5% (77 of 134) of the melanoma specimens analysed (a representative MDR1/P-gp positive tumour is shown in Figure 1A). Specific staining was localised to cell membrane/cytoplasm. Approximately 43% of tumours did not express MDR1/P-gp protein. MDR1/P-gp expression in primary and metastatic tumours, when analysed as separate cohorts, was 50 and 73.8%, respectively.

### MRP-1 expression

MRP-1-specific staining was observed in 67.2% (90 per 134) of the tumours analysed ((Figure 1B) illustrates a typical example). In all 33% of all tumours did not express MRP-1 protein. Independent assessment of the primary tumours and metastases indicated MRP-1 to be detected in 56.5 and 90.5% of cases, respectively.

### MDR1/P-gp and MRP-1 co-expression

The expressions of MDR1/P-gp and MRP-1 proteins were significantly associated (*P*=0.007). The majority (80.6%) of melanomas expressed at least one of the efflux pumps analysed; 44% expressed both, while only 19.4% expressed neither MDR1/P-gp nor MRP-1 proteins (see Table 2a). For the primary tumour cohort alone, as summarised in Table 2b, approximately 73% of cases expressed at least one of these pumps, 27% had neither MDR1/P-gp nor MRP-1, while 34% expressed both proteins. Table 2c indicates that the majority (97.6%) of the metastatic specimens expressed MDR1/P-gp and/or MRP-1; 67% had both. Almost a quarter (23.8%) of metastatic tumours had MRP-1, but lacked MDR1/P-gp expression, whereas only 7% expressed MDR1/P-gp alone.

Overall, as shown in Table 3, a direct comparison of MDR1/P-gp and MRP-1 efflux pump expression in the primary *vs* metastatic specimens indicated a significantly higher percentage of metastatic tumours, when compared with primary tumours, expressed MDR1/P-gp (primary 50% metastases 74% *P*=0.010) and MRP-1 (primary 57% metastases 91% *P*⩽0.0001), with MRP-1 being the most prevalent efflux pump in these specimens.

### MDR1/P-gp and MRP-1 expression in relation to clinicopathological features

Using *χ*^*2*^ analysis of the 134 patients as a whole (summarised in Table 4), MDR1/P-gp expression was found to be significantly (*P*=0.013) associated with lymph node positivity and tended to be associated with increased Breslow's depth; however, the latter case, showed statistical significance (*P*=0.052). MRP-1 expression was also significantly associated with spread to the lymph nodes (*P*<0.0005) and with more extensive invasion of the melanoma, i.e., when comparing MRP-1 expression in specimens with Clark's level II, III and IV vs Clark's level V; *P*=0.032 (incidentally, comparison of MRP-1 expression in those specimens with Clark's levels II and III with that in specimens with Clark's levels IV and V also supported the association between MRP-1 expression and increasing stages of disease; (although NS, *P*=0.051)).

Assessing the expression of these efflux pumps in the cohort of primary tumours alone (Table 5) showed similar trends to those seen when evaluating all 134 cases, that is, MDR1/P-gp expression being associated with spread to the lymph nodes (*P*=0.049) and increasing Breslow's depth (although NS, *P*=0.056), with MRP-1 expression being significantly associated with increasing Clark's level (*P*=0.023).

In relation to the metastases cohort alone, as the majority of these specimens expressed MRP-1 (∼91%) and MDR1/P-gp (∼74%), seeking association with clinicopathological factors is not relevant here.

## Discussion

Malignant melanoma is one of the most challenging cancers to treat, resulting in highest per-death loss of years of potential life expectancy with the exception of adult leukaemia (Seetharamu *et al*, 2009). Unfortunately, the effectiveness of chemotherapeutic regimes evaluated in this cancer type is compromised and very limited, owing to the yet undefined highly resistant nature of this cancer.

Drug efflux pumps, MDR1/P-gp and MRP-1, have been associated with chemo-resistance in a number of cancer types. Studies of drug efflux pumps in melanoma have, however, been very limited to date. As outlined previously, studies of melanoma cell line models have resulted in conflicting results, some suggesting a role for MRP-1 (Berger *et al*, 1997), while others propose MDR1/P-gp to be potentially of more relevance (Molinari *et al*, 2000; Colone *et al*, 2008). Both earlier studies of melanoma tissue specimens (from 8 and 58 patients, evaluating mRNAs and proteins, respectively) reported MRP-1, but generally not MDR P-gp, expression (Schadendorf *et al*, 1995; Ichihashi and Kitajima, 2001). Although those studies were important in highlighting the potential relevance of a drug efflux pump (namely, MRP-1) in melanoma, the numbers of specimens included were very limited. Furthermore, associations with patients' clinical characteristics were not investigated.

Here, in this more extensive study of 134 melanoma specimens, in agreement with the studies by Schadendorf *et al* (1995), we report MRP-1 protein to be typically expressed in melanoma. However, whereas Schadendorf *et al* (1995) reported the prevalence of expression to be the same in primary tumours and metastases, we observed that significantly higher percentages of metastatic specimens, compared with primary tumours, express MRP-1. Furthermore, in contrast to the work reported by Schadendorf *et al* (1995) in which MDR1/P-gp was detected in only 1 of 21 primary specimens and was absent from metastases, we detected MDR1/P-gp in more than half of the specimens analysed. As for MRP-1, expression of MDR1/P-gp was significantly more frequent in metastases compared with primary tumours. The discrepancies between our work and that reported by Schadendorf *et al* (1995) may, at least partly, be related to the larger cohort of specimens to which we had access in our analysis. We propose that such large studies could potentially contribute to more clinically relevant results.

This, we believe, is the first study evaluating potential associations between MDR1/P-gp and/or MRP-1 expression and melanoma patients' clinicopathological characteristics. Although we found no significant association between either the presence of either efflux pump or the age and gender of the patients, we identified that expression of these pumps tended to be associated with the more aggressive nature of melanoma. Specifically, MRP-1 expression was significantly associated with tumours classified as having a Clark's level of V (*i.e.,* invading the subcutaneous fat, instead of being limited to the epidermal or dermal layers of the skin). Although statistical significance was not reached, MDR1/P-gp expression tended to be more strongly associated with tumours having a staging Breslow's thickness of >4 mm which, according to the AJCC guidelines, is prognostic of a 5-year. survival of approximately 50% (compared with 95–100% for <1 mm; 80–95% for 1–2 mm; and 60–80% for 2.1–4 mm). Furthermore, we established that both MRP-1 and MDR1/P-gp expression are significantly associated with spread of cancer to the lymph nodes. The observed associations between expression of these efflux pumps and the more aggressive phenotype of melanoma, together with the significantly higher prevalence of these pumps (MRP-1, in particular) in metastases compared with primary specimens, suggests that MRP-1 and MDR1/P-gp may contribute, at least to some extent, to the incurable nature of malignant melanoma.

In conclusion, this study indicates that expression of drug efflux pumps, MRP-1 and MDR1/P-gp, is common in melanoma, especially in metastases, and so potentially could contribute to the chemo-resistant biology and thus incurable nature of this cancer type. The expression of these proteins – and, in particular, the predominant expression of MRP-1 – suggests that these pumps may be important contributors to this chemo-resistance. These findings suggest that the inhibition of multiple drug efflux pumps might be necessary if clinically relevant MDR reversal is to be achieved in malignant melanoma.

## Figures and Tables

**Table 1:** Clinicopathological features of patients studied for MDR1/P-gp and MRP-1 expression in melanoma primary and metastatic specimens

**Characteristics**	**Primary (%)**	**Metastatic (%)**
*Age (years)*		
<59	45.6 (42/92)	54.8 (23/42)
>59	54.3 (50/92)	45.2 (19/42)
		
*Gender*		
Male	32.6 (30/92)	50.0 (21/42)
Female	67.4 (62/92)	50.0 (21/42)
		
*Lymph node spread*		
Negative	92.4 (85/92)	28.6 (12/42)
Positive	7.6 (7/92)	71.4 (30/42)
		
*Clark's level*		
I	1.08 (1/92)	97.6 (41/42)
II	10.9 (10/92)	0 (0/42)
III	32.3 (30/92)	0 (0/42)
IV	22.8 (21/92)	0 (0/42)
V	6.5 (6/92)	2.4 (1/42)
Unknown	26.1 (24/92)	0 (0/42)
		
*Breslow thickness*		
<1 mm	31.5 (29/92)	2.4 (1/42)
1–2 mm	18.5 (17/92)	2.4 (1/42)
2.1–4 mm	10.9 (10/92)	0 (0/42)
>4 mm	21.7 (20/92)	4.8 (2/42)
Unknown	17.4 (16/92)	90.5 (38/42)
		
*Relapse*		
Relapse ⩽5 years	25.0 (23/92)	19.0 (8/42)
Did not Relapse ⩽5 years	14.1 (13/92)	7.1 (3/42)
Unknown	60.9 (56/92)	73.8 (31/42)

**Table 2:** Comparison of MDR1/P-gp and MRP-1 co-expression in (a) all melanoma specimens analysed; (b) primary melanoma specimens only; and (c) metastatic melanoma specimens only

**Drug efflux pump**	**% Cases**
*(a)*	
MRP1^+^and MDR1/P-gp^+^	44.0 (59/134)
MRP1^−^ and MDR1/P-gp^+^	13.4 (18/134)
MRP1^+^ and MDR1/P-gp^−^	23.1 (31/134)
MRP1^−^ and MDR1/P-gp^−^	19.4 (26/134)
	
*(b)*	
MRP1^+^ and MDR1/P-gp^+^	33.7 (31/92)
MRP1^−^ and MDR1/P-gp^+^	16.3 (15/92)
MRP1^+^ and MDR1/P-gp^−^	22.8 (21/92)
MRP1^−^ and MDR1/P-gp ^−^	27.2 (25/92)
	
*(c)*	
MRP1^+^ and MDR1/P-gp^+^	66.7 (28/42)
MRP1^−^ and MDR1/P-gp^+^	7.1 (3/42)
MRP1^+^ and MDR1/P-gp^−^	23.8 (10/42)
MRP1^−^ and MDR1/P-gp^−^	2.4 (1/42)

Abbreviations: MDR1/P-gp=multi-drug resistance; MRP=multidrug resistance-associated protein.

**Table 3:** Correlation between expression of MDR1/P-gp and MRP-1 protein in primary *vs* metastatic specimens

**Tumour site**	**No. of cases**	**MDR P-gp (%)**	***P***	**No. of cases**	**MRP-1 (%)**	***P***
Primary	46/92	50	0.010^*^	52/92	56.5	*P*<0.000^*^
Metastasis	31/42	73.8		38/42	90.5	

Abbreviations: MDR1/P-gp=multi-drug resistance; MRP=multidrug resistance-associated protein.

*P* values from *χ*^2^ analyses; ^*^indicates significant parameter.

**Table 4:** Correlation between clinicopathological factors and expression of MDR1/P-gp and MRP-1 protein in all melanoma specimens analysed

**Characteristics**	**No. of cases**	**MDR P-gp (%)**	***P***	**No. of cases**	**MRP-1 (%)**	***P***
*Age (years)*						
<59	34/65	52.3	0.241	43/65	66.2	0.809
⩾59	43/69	62.3		47/69	68.1	
						
*Gender*						
Male	30/51	58.8	0.803	39/51	76.5	0.072
Female	47/83	56.6		51/83	61.4	
						
*Lymph node spread*						
Negative	50/98	51.0	0.013^*^	57/98	58.2	<0.0005^*^
Positive	27/36	75.0		33/36	91.7	
						
*Clark's level*						
II and III	20/41	48.8	0.251	19/41	46.3	0.051
IV and V	17/27	63.0		19/27	70.3	
						
*Clark's level*						
II, III, IV	32/62	51.6	0.136	32/62	51.6	0.032^*^
V	5/6	83.3		6/6	100	
						
*Breslow thickness*						
<1 mm	14/30	46.7	0.052	13/30	43.3	0.107
1–4 mm	11/28	39.3		16/28	57.1	
>4 mm	16/22	72.7		11/22	50.0	
						
*Relapse*						
Relapse within 5 years	17/31		0.260	18/31		0.252

Abbreviations: MDR1/P-gp=multi-drug resistance; MRP=multidrug resistance-associated protein. *P* values from *χ*^2^ analyses; ^*^indicates significant parameter; Clark's score – information was available for 68 of 134 cases; Breslow's thickness – information was available for 80 of 134 cases.

**Table 5:** Correlation between clinicopathological factors and expression of MDR1/P-gp and MRP-1 protein in primary tumours only

**Characteristics**	**No. of cases**	**MDR P-gp (%)**	***P***	**No. of cases**	**MRP-1 (%)**	***P***
*Age (years)*						
<59	19/42	45.2	0.402	22/42	52.4	0.463
⩾59	27/50	54.0		30/50	60.0	
						
*Gender*						
Male	14/30	46.7	0.656	21/30	70.0	0.070
Female	32/62	51.6		31/62	50.0	
						
*Lymph node spread*						
Negative	40/85	47.1	0.049^*^	46/85	54.1	0.105
Positive	6/7	85.7		6/7	85.7	
						
*Clark's level*						
II and III	20/41	48.8	0.251	19/41	46.3	0.051
IV and V	17/27	63.0		19/27	70.3	
						
*Clark's level*						
II, III, IV	32/62	51.6	0.136	32/62	51.6	0.023^*^
V	5/6	83.3		6/6	100.0	
						
*Breslow thickness*						
and lt;1 mm	14/29	48.3	0.056	12/29	41.4	0.139
1–4 mm	11/27	40.7		15/27	55.6	
>4 mm	15/20	75.0		14/20	70.0	
						
*Relapse*						
Relapse within 5 years	11/23	47.8	0.587	10/23	43.5	0.137

Abbreviations: MDR1/P-gp=multi-drug resistance; MRP=multidrug resistance-associated protein. *P* values from *χ*^*2*^ analyses; ^*^indicates significant parameter; Clark's score – information was available for 68 of 92 cases; Breslow's thickness – information was available for 76 of 92 cases; relapse information available for 36 cases.
